# Announcing the availability of oral HIV self-test kits via text message to increase HIV testing among hard-to-reach truckers in Kenya: a randomized controlled trial

**DOI:** 10.1186/s12889-018-6345-1

**Published:** 2019-01-03

**Authors:** Elizabeth A. Kelvin, Gavin George, Samuel Kinyanjui, Eva Mwai, Matthew L. Romo, Faith Oruko, Jacob O. Odhiambo, Eston N. Nyaga, Joanne E. Mantell, Kaymarlin Govender

**Affiliations:** 1 Department of Epidemiology and Biostatistics, CUNY Graduate School of Public Health and Health Policy, City University of New York, 55 West 125th Street, New York, NY 10027 USA; 2 CUNY Institute for Implementation Science in Population Health, City University of New York, 55 West 125th Street, New York, NY 10027 USA; 3 Health Economics and HIV and AIDS Research Division, University of KwaZulu-Natal, Westville Campus, J block, Level 4, University Road, Durban, South Africa; 4 North Star Alliance, PO Box 165, Nairobi, 00202 Kenya; 5 HIV Center for Clinical and Behavioral Studies, Department of Psychiatry, Division of Gender, Sexuality and Health, New York State Psychiatric Institute & Columbia University, 1051 Riverside Drive, Unit 15, New York, NY 10032 USA

**Keywords:** HIV, Self-testing, Kenya, Truckers, Text message, Randomized controlled trial

## Abstract

**Background:**

Truckers in sub-Saharan Africa are at higher risk of contracting HIV than the general population. HIV self-testing may be a way to increase testing rates in this high-risk population. The objective of this randomized controlled trial was to assess whether informing truckers who do not test for HIV regularly about the availability of HIV self-testing kits at roadside wellness centers in Kenya using text messages would increase HIV testing rates compared to the current program in which they are sent text messages about the availability of HIV testing in general.

**Methods:**

A sample of 2262 male truckers registered in the North Star Alliance electronic health record system who, based on these records, were not testing for HIV regularly were randomized to one of three study groups in which they were sent text messages about the availability of (1) oral HIV self-test kits at all 8 North Star Alliance Kenya clinics that was sent three times (intervention), (2) HIV testing in general (not self-testing) at all North Star Alliance clinics sent three times (enhanced standard of care [SOC]), or (3) HIV testing in general (not self-testing) at all North Star Alliance clinics sent one time (SOC). We looked at HIV testing over a 2-month study period following the first text.

**Results:**

Truckers in the intervention group were significantly more likely to test for HIV compared to those in the enhanced SOC (OR = 2.7, *p* = 0.009). There was no difference in HIV testing between those in the enhanced SOC and the SOC groups. Of those in the intervention group who tested, 64.5% chose the self-test and 35.5% chose the standard provider-administered blood-based HIV test. Although the intervention more than doubled HIV testing rates, because HIV testing rates were so low in this population (by design as we selected irregular testers), even in the intervention group more than 96% of participants did not test.

**Conclusions:**

Announcing the availability of HIV self-testing via text message increased HIV testing rates among truckers who were not regularly accessing HIV testing. However, self-testing is only a partial solution to increasing testing rates in this hard to reach population.

**Trial registration:**

This trial was registered prior to enrollment at the Registry for International Impact Evaluations (RIDIE STUDY ID: 582a2462ae2ab): http://ridie.3ieimpact.org/index.php?r=search/detailView&id=492. It was also registered after completion at ClinicalTrials.gov (ClinicalTrials.gov Identifier: NCT03662165): https://clinicaltrials.gov/ct2/show/NCT03662165?term=NCT03662165&type=Intr&cond=HIV&rank=1.

## Background

Truckers in ub-Saharan Africa are at high risk for HIV due to high levels of risk behavior. A study in 1991 among a sample of truckers in Kenya found 61% reported having visited sex workers, only 32% had ever used condoms [1], and 18% tested HIV-positive [2]. A 2015 study among truckers in Kenya found that 56% had paid for sex in the past 6 months, 47% had a regular partner along their trucking route in addition to a wife or girlfriend at home, and only 14% had always used condoms during sex in the past 6 months [3]. Studies among truckers in other countries have found high HIV prevalence, including 23% in Nigeria [4], 15.4% in Mozambique [5], and 26% in South Africa [6]. Because truckers cross national and international borders for work, high HIV prevalence among truckers can lead to HIV spread across these borders as well as from casual and commercial partners along trucking routes to wives and main partners at home [7, 8].

Few studies have looked at HIV testing among truckers. One study among 1881 truckers in South Africa in 2003–2004 found that only 38.2% had ever been tested for HIV [6]. HIV self-testing might be a more acceptable and convenient HIV testing option for truckers. In a 2015 study [3, 9], 305 truckers were recruited from the waiting room of two roadside wellness clinics in Kenya, randomized to be offered either a provider-administered blood (finger-prick) HIV test (Standard of Care [SOC]), or an oral HIV self-test as an option in addition to the standard HIV test. Those in the intervention group were also allowed to pick-up self-test kits 3–6 months later. In this study, HIV testing rates were higher in the intervention group the first time HIV testing was offered immediately following randomization (Odds Ratio [OR] = 2.8, *p* = 0.002), but not during the 6-month follow-up (OR = 1.0, *p* = 0.972). The reason for this discrepancy could be that the self-test increases testing when it is offered to someone who is already in the clinic, but it is not sufficient motivation to bring people to the clinic to get a test kit.

A number of studies among other population groups have found that direct distribution of HIV self-test kits can increase testing rates. A study in Kenya that gave pregnant women self-testing kits to take home to their male partners found a 1.6 times higher testing rate among those partners compared to when women gave their partners an invitation card to come to the clinic for HIV testing (*p* < 0.001) [10]. A similar study in Uganda had a similar impact (risk ratio [RR] = 2.1) [11], and two studies in Zambia and Uganda that offered self-test kits to female sex workers through peer educators found higher testing rates compared to the SOC (referral to a clinic for testing), although the difference was only significant for the Uganda study (RR = 1.3, *P* < 0.001 in Uganda and RR = 1.1, *P* = 0.11 in Zambia) [12, 13]. A home-based (door-to-door) HIV testing study in Zambia found that offering a self-test in addition to the standard provider-administered HIV test increased testing rates from 55.1 to 60.4% [11].

However, direct distribution may be unfeasible in some situations, especially for truckers who are mobile and have unpredictable schedules. Therefore, we conducted a randomized controlled trial to assess whether alerting truckers via text message about the availability of HIV self-testing at clinics located at major transit hubs in Kenya would increase HIV testing rates. We conducted a similar study among female sex workers in Kenya and found that those who were notified about the availability of HIV self-testing via text message were significantly more likely to come to a clinic for HIV testing (OR 1.9, *p* = 0.001) compared to those who received text messages about HIV testing in general (not about self-testing) [14]. For this study, we used similar methods and specifically targeted truckers who were not already accessing HIV testing regularly to see if alerting them about the availability of HIV self-testing would increase HIV testing rates among these hard-to-reach truckers.

## Methods

For this study, we used methods similar to those we used in the study among female sex workers in Kenya [14]. Below we briefly describe the main design characteristics of the study.

### Setting and population

This study was conducted in the North Star Alliance clinic system, which aims to bring primary and secondary health services to hard-to-reach groups across Africa, including truckers and sex workers. In 2015, the North Star Alliance reported 253,227 client-visits at their 36 roadside wellness clinics across Africa, which includes the eight clinics in Kenya participating in this study. Eighteen percent of these visits included HIV testing [15]. Information on clinic clients is entered into the electronic health record system (EHRS), including their mobile phone number if the client has one and is willing to provide it.

### Current HIV testing standard of care

Clients presenting at North Star Alliance clinics are offered a blood-based (finger-prick), provider-administered rapid HIV test (standard HIV test). HIV testing is tracked in the EHRS and text message reminder is sent to those clients who have not tested in the past three months. The message reads “North Star Alliance East Africa would wish to kindly remind you to visit any of our roadside wellness centers for HIV testing. Your health, our priority.”

### Sample and eligibility criteria

Study participants were selected from the EHRS. Eligibility criteria included: (a) no indication of being HIV-positive, (b) Kenya resident, (c) valid mobile phone number listed, (d) had fewer than four HIV tests in the past year (suggesting that they were not testing every three months as recommended for those at high risk [16]), (e) no indication of an HIV test in the past three months, (f) had not participated in our previous study on self-administered HIV testing [3, 9, 17], (g) were male, and (h) worked as truckers, including drivers and assistants (turn boys).

Once the sample of eligible participants was selected, the North Star Alliance sent a passive consent text message twice, a week apart, first Kiswahili and then in English that informed clients that their medical record data would be used for program evaluation and provided instructions for how clients could opt out of being included if they chose to do so. Clients who indicated they wanted to opt out of the evaluation were removed from the sample prior to randomization. Requests to opt out after randomization were considered study drop-outs and were excluded from the data analysis.

### Randomization and intervention

The eligible individuals who remained in the sample after the consent process were randomized to one of three study groups.

1. Intervention, in which a text message was sent three times, one week apart, alternating in English or Kiswahili that stated:
  “You can now self-test at home or in the clinic for HIV using a new test kit available from all North Star Alliance clinics in Kenya. Your health, our priority.”
2. Enhanced SOC, in which the SOC text previously described was sent three times, alternating in English or Kiswahili.
3. SOC in which the SOC text was sent one time in both Kiswahili and English concurrently.

### Sample size and power

We powered our study for to compare the HIV testing rates over the two months following the first text message between the intervention and enhanced SOC groups. We determined the required sample size for these two groups using data from the EHRS, which suggested that the HIV testing rates after sending the SOC text message among truckers was ≤68%. We used the maximum rate of 68% in order to account for any small increase that might occur when sending the text three times instead of only once in the enhanced SOC. In order to detect a 20% increase in the proportion testing in the intervention group over the anticipated 68% in the enhanced SOC group (RR = 1.2, OR = 1.4) at 80% power and 95% confidence level, we required a sample size 750 truckers in each of the two groups. Therefore, we set our target sample size to 750 truckers in the intervention and 750 in the enhanced SOC. If we had > 1500 eligible clients, we planned to randomize the extra in the sample to the SOC group. Thus the probability of being randomized into each of the three study groups was determined by the number of eligible participants in order to ensure 750 in the intervention and 750 in the enhanced SOC groups. It turned out that we had 2262 eligible clients participating, giving us a randomization ratio of 1:1:1.02 for the intervention, enhanced SOC and SOC groups respectively.

### Masking & HIV testing procedures (program)

Participants were not informed about the specific research question or that they would be randomized to different HIV testing programs in order to avoid bias. Participants in both SOC groups (enhanced or standard) were offered only the standard HIV test when seeking clinic services. Those in the intervention group who sought services in one of the clinics in Kenya were given a demonstration of the self-testing kit and offered choices among (1) the SOC HIV test, (2) the self-administered oral HIV test for use in the clinic with provider supervision, or (3) a self-administered oral HIV test kit to take and use outside of the clinic (e.g. home use).

Those who accepted the standard HIV test underwent the standard pre- and post-testing counselling and testing process. Those who chose the supervised self-test in the clinic received pretest counseling and then were given the OraQuick HIV self-test kit [13] to use while a counselor sat in the room to answer questions and provide guidance if needed, followed by posttest counseling. Those who took a self-test kit home were given pre-test counseling in the clinic and then instructed to use their test kit within three days and to call or send a text message if they had any questions while testing and after using the test to receive a call-back for post-test counseling and referrals. Participants who did not contact the clinic staff by day three were called repeatedly by clinic staff until they were reached.

The study was approved by the City University of New York (CUNY) Institutional Review Board, the Kenya Medical Research Institute Ethics Committee (KEMRI), and the University of KwaZulu-Natal Biomedical Research Ethics Committee (BREC).

### Data analysis

We described the sample overall and by group to ensure that the randomization worked as expected. The statistical significance of any differences by group was assessed using the chi-square test for categorical variables and the Kruskal Wallis test for numeric variables. We compared the proportion who tested for HIV during the two-month follow-up period between clients in the intervention and those in the enhanced SOC group (primary comparison) as well as between those in the enhanced SOC and those in the SOC group (secondary comparison) to estimate the impact of the text message content (i.e. about self-testing kits or about HIV testing in general) and number (one versus three) on HIV testing, respectively. We also looked at differences in clinic contact for any reason (i.e. the client came to a North Star Alliance clinic during the follow-up period for any services, not just HIV testing) between the groups to determine if the text message influenced how many truckers came to the clinics even if some of them did not test. We used logistic regression for these comparisons and all models were based on intent-to-treat; even if someone did not receive the text messages we sent, they were included in the arm to which they were randomized.

EHRSs are not a perfect data source and we found that there were 5 clients without an indication of HIV testing in the EHRS who were documented as having self-tested in the administrative records kept by the clinics to track the use of self-test kits for inventory purposes and to follow-up with those who took self-test kits for home-use. We considered the EHRS data as our primary data source, since we did not have administrative data on non-self-testers, and first analyzed the data as reported in the EHRS. However, as a sensitivity analysis we also ran the models coding those five participants as having tested, as documented in the administrative records, to determine if our results changed substantially.

## Results

### Description of the sample

**Fig. 1:**
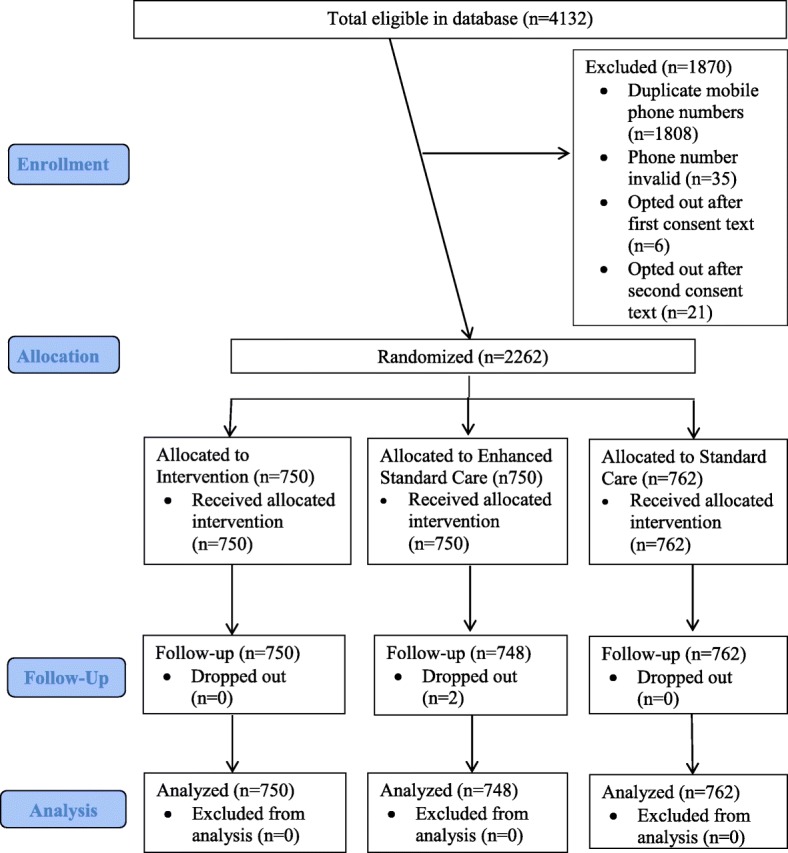
Flow of male truck driver participants (Consort Flowchart)

On December 13, 2016, we selected a sample of 4132 trucker clients from the North Star Alliance EHRS who met the study eligibility criteria. After we deleted duplicate phone numbers from the sample so as not to send the messages to the same phone multiple times, we were left with a sample of 2324 truckers to whom we sent the first consent text message, after which 6 opted out. A week later we sent the second consent text message, after which an additional 21 opted out. We also excluded 35 phone numbers that were returned as invalid. The first study texts were sent on Dec 20, 2016 to 2262 truckers. During the follow-up period, two additional truckers contacted us to opt out and were excluded from analysis as study drop-outs. (Fig. 1).

The mean age of the participants was 35.3 years and 76.3% were married or living with a partner. Most participants were drivers (84.9%) and 15.1% were assistants. Overall, 73.1% had not had an HIV test in the past year and, among those who had tested in the past year, the mean time since testing was 5.9 months. There were no significant differences in these characteristics by study group. (Table 1).

**Table 1:** Descriptive statistics for the sample overall and by the three study groups

	Total	Intervention	Enhanced SOC	SOC	*P*-value
Total, n (total %)	2260 (100.0%)	750 (33.2%)	748 (33.1%)	762 (33.7%)	NA
Male, n (total %)	2260 (100.0%)	750 (33.2%)	748 (33.1%)	762 (33.7%)	NA
Age					0.590^1^
Mean (SD)	35.3 (8.7)	35.2 (8.9)	35.5 (8.6)	35.2 (8.5)	
Median (Range)	34.0 (18.0–76.0)	34.0 (18.0–75.0)	34.5 (18.0–76.0)	34.0 (18.0–68.0)	
Marital status					0.561
Married/Cohabitating	1725 (76.3%)	569 (75.9)	581 (77.7%)	575 (75.5%)	
Unmarried (single, divorced/separated)	535 (23.7%)	181 (24.1%)	167 (22.3%)	187 (24.5%)	
Trucker Job					0.859
Driver	1917 (84.9%)	633 (84.4%)	634 (84.9%)	650 (85.4%)	
Assistant (Turnboy)	341 (15.1%)	117 (15.6%)	113 (15.1%)	111 (14.6%)	
Missing	2	0	1	1	
Test in past year, n (column %)					0.254
Yes	607 (26.9%)	199 (26.5%)	188 (25.1%)	220 (28.9%)	
No	1653 (73.1%)	551 (73.5%)	560 (74.9%)	542 (71.1%)	
Months since last test among those tested in past year (among those who tested in past year)					0.301
Mean (SD)	5.9 (2.6)	5,8 (2.5)	6.1 (2.7)	5.8 (2.6)	
Median (Range)	4.0 (3.0–11.0)	4.0 (3.0–11.0)	5.0 (3.0–11.0)	4.0 (3.0–11.0)	
North Star Alliance Clinic where last seen, n (column %)					0.582^2^
Burnt Forest, Kenya	17 (0.8%)	7 (0.9%)	5 (0.7%)	5 (0.8)	
Emali, Kenya	176 (7.8%)	53 (7.1%)	57 (7.6%)	66 (8.7%)	
Jomvu, Kenya	1094 (48.4%)	363 (48.4%)	370 (49.5%)	361 (47.4%)	
Maai Mahiu, Kenya	5 (0.2)	3 (0.4%)	1 (0.1%)	1 (0.1%)	
Mlolongo, Kenya	20 (0.9%)	4 (0.5%)	9 (1.2%)	7 (0.9%)	
Mombasa, Kenya	146 (6.5%)	45 (6.0%)	57 (7.6%)	44 (5.8%)	
Namanga, Kenya	174 (7.7%)	54 (7.2%)	51 (6.8%)	69 (9.1%)	
Salgaa, Kenya	625 (27.7%)	220 (29.3%)	197 (26.3%)	208 (27.3%)	
Malaba, Uganda	1 (0.0%)	0 (0.0%)	0 (0.0%)	1 (0.1%)	
Katuna, Uganda	1 (0.0%)	0 (0.0%)	1 (0.1%)	0 (0.0%)	
Tunduma, Tanzania	1 (0.0%)	1 (0.1%)	0 (0.0%)	0 (0.0%)	

^1^P-value from Kruskal Wallis Test

^2^P-value from Chi-square test

Logistic regression models comparing those in the intervention group to those in the enhanced SOC group.

Ten (1.3%) truckers in the enhanced SOC group and 26 (3.5%) in the intervention group tested for HIV during the study period. Those in the intervention group had 2.7 times greater odds of testing compared to those in the enhanced SOC group, which was statistically significant (*p* = 0.009). If we include the additional 5 clients who had an indication of HIV self-testing in the administrative records as having HIV tested, the odds ratio increased to 3.2 (*p* = 0.002). (Table 2).

**Table 2:** Logistic regression model results for HIV testing and clinic contact comparing the intervention to the enhanced SOC groups

	Total, n (%)	Enhanced SOC group, n (%)	Intervention group, n (%)	OR (95% CI)	Chi-Square p-value
Total	1498 (100.0%)	748 (49.9%)	750 (50.1%)	NA	NA
Tested for HIV (according to EHRS only)					
Yes	36 (2.4%)	10 (1.3%)	26 (3.5%)	2.7 (1.3–5.5)	0.009
No	1462 (97.6%)	738 (98.7%)	724 (96.5%)		
Tested for HIV (including those with an HIV test indication only in the clinic administrative records)^a^					
Yes	41 (2.7%)	10 (1.3%)	31 (4.1%)	3.2 (1.6–6.5)	0.002
No	1457 (97.3%)	738 (98.7%)	719 (95.9%)		
Received any clinic services (according to EHRS only)					
Yes	169 (11.3%)	80 (10.7%)	89 (11.9%)	1.1 (0.8–1.6)	0.474
No	1329 (88.7%)	668 (89.3%)	661 (88.1%)		
Received any clinic services (including those with an HIV test indication only in the clinic administrative records)^a^					
Yes	174 (11.6%)	80 (10.7%)	94 (12.5%)	1.2 (0.9–1.6)	0.267
No	1324 (88.4%)	668 (89.3%)	656 (87.5%)		

^a^5 clients were noted as having HIV tested in the clinic administrative records used for tracking self-test kits and posttest counseling but their test was not indicated in the EHRS. Here these 5 are recoded (data cleaned based on additional information) as having HIV-tested and the analysis rerun

Eighty (10.7%) truckers in the enhanced SOC group and 89 (11.9%) in the intervention group had some form of clinic contact during the study period. Those in the intervention group had 1.1 times greater odds of clinic contact compared to those in the enhanced SOC group, but the difference was not statistically significant (*p* = 0.474). If we include the additional 5 clients who had an indication of HIV self-testing in the administrative records, the odds ratio increased to 1.2 (*p* = 0.267). (Table 2).

### Logistic regression models comparing those in the enhanced SOC group to those in the SOC group

Overall 1.3% of clients in the enhanced SOC group and 1.3% in the SOC group tested for HIV during the study period (OR = 1.0, *p* = 0.967). There was also no difference in clinic contact between the two groups (OR = 1.0, *p* = 0.987). (Table 3).

**Table 3:** Logistic regression model results for HIV testing and clinic contact comparing the enhanced SOC to the SOC groups

	Total, n (%)	SOC group, n (%)	Enhanced SOC group, n (%)	OR (95% CI)	Chi-Square p-value
Total with	1510 (100.0%)	762 (50.5%)	748 (49.5%)	NA	NA
Tested for HIV (According to EHRS)					
Yes	20 (1.3%)	10 (1.3%)	10 (1.3%)	1.0 (0.4–2.5)	0.967
No	1490 (98.7%)	752 (98.7%)	738 (98.7%)		
Received any clinic services (According to EHRS)					
Yes	161 (10.7%)	81 (10.6%)	80 (10.7%)	1.0 (0.7–1.4)	0.967
No	1349 (89.3%)	681 (89.4%)	668 (89.3%)		

### HIV testing choices among those in the intervention group

Of the 31 truckers who tested in the intervention group, including the 5 from the administrative records, 20 (64.5%) chose to self-test. Of those, 14 (70.0%) chose to self-test in the clinic with supervision, 5 (25.0%) chose to take a test kit for home use, and one (5.0%) client initially took a test kit for home use but then changed his mind and returned to the clinic to test with supervision. (Table 4).

**Table 4:** Description of HIV testing choices made for those in the intervention group who tested ^a^

	Study Sample
Total testers in the intervention group	31 (100.0%)
Chose a self-test	
Yes	20 (64.5%)
No	11 (35.5%)
Self-testing method chosen among those who self-tested	
In the clinic with counselor supervision	14 (70.0%)
Took test kit for home use	5 (25.0%)
Took test kit for home use but returned to the clinic to use it with supervision	1 (5.0%)
Contacted clinic during or after self-testing at home	
Called while testing with questions	2 (33.3%)^b^
Called after testing for posttest counseling	1 (16.7%)
Called both during and after testing	2 (33.3%)
Did not call	1 (16.7%)
Number of contact attempts made by counselor to reach the client who took self-test kit but did not call	2

^a^Includes the 5 clients who were listed as self-testers in the administrative records but not in the EHRS

^b^Includes the one client who decided to return to the clinic to self-test after initially taking the test-kit for home use

Of the 6 who took a test kit for home use (including the one who changed his mind), 4 (66.7%) called the counselor while testing with questions, and 3 (50.0%) called after testing for posttest counseling (two called both during and after the testing). One client did not call and it took two attempts for the counselor to reach him to learn that he had used the test, with the counselor proceeding to provide posttest counseling. (Table 4) Five participants tested HIV+ during the study, all of whom were in the intervention group. (Data not shown).

## Discussion

HIV testing in this sample of hard-to-reach truckers was very low, at 1.3% in both the enhanced SOC and the SOC groups during the study period. Texting about the availability of HIV self-test kits increased HIV testing rates to 3.5% (OR = 2.7, or 4.1%, OR = 3.2 when including the administrative record data). This increase in testing rate is not very different from the study that recruited truckers from the waiting room of two North Star Alliance clinics in Kenya (OR = 2.8) [3, 9]. However, participants in the previous trucker study were testing at much higher rates than those in the current study, in part by design as we purposely selected irregular testers in this study. Thus, the relative increase in testing rate was fairly consistent across the two trucker studies that differed in terms of how the self-test was initially introduced (by the counselor when the client was already in the clinic versus via text message) and in terms of the HIV testing history of the participants. However, the absolute difference in testing rates varied widely between the two groups (2.2 per 100 more truckers testing in this study versus 14.9 per 100 truckers in the previous study) because the baseline (or SOC) testing rate in this study was so much lower. It is important to note that, in this study, five HIV-infections were identified in the intervention group. This may suggest that the intervention encouraged some to test who were not going to test in the SOC program, and that these people were more likely to be HIV-positive, either because they had not tested in a long time, if ever, and thus the pool of unidentified infections among those testing in the intervention group was greater than that among those testing in the SOC groups, or because those at higher risk were more likely to test under the intervention than the SOC. As participants were randomized to the three study arms, there is no reason to suspect that this higher rate of HIV-positive results in the intervention group was due to systematic differences in the participants in that study arm compared to the other two arms, although with chance, it is possible. It is also unlikely that the rate of false positives was higher among those in the intervention group, as the specificity of the self-test kit is actually slightly higher (99.9%) [18] than that of the SOC test (98.5%) [19].

Importantly, our study found that announcing the availability of HIV self-test kits via text did result in more clients coming to the clinics to access these kits and direct distribution is not the only effective distribution model. Our study among female sex workers who were irregular HIV testing also found that the text alert about the availability of HIV self-test kits increased testing rates (OR 1.9, *p* = 0.001) [14]. Thus if this basic text-message alert about HIV self-test kit availability worked among our hard-to-reach, high risk groups such as truckers and sex workers who are not accessing HIV testing regularly, it may work even better in the general population. Use of text messaging to announce the availability of HIV self-testing could be an easy and cost-effective strategy to increase uptake as countries roll out self-testing. HIV self-testing was approved in Kenya in May 2017, a few months after the completion of our study, with test kits being made available for a cost of around $8 US [16]. Text messages might be a useful mechanism to inform the public about where self-test kits can be obtained as they become available in new locations. Due to the character limits, the text messages we sent were fairly simple; however, videos depicting the self-testing process and package inserts are now available online in multiple African languages, including Kiswahili [20]. With the proliferation of smart phones in Kenya [21], links to demonstrations of the self-testing process could be sent to people so they can see what the self-test is and how it works. Providing more information about the self-test might further increase in test uptake over what we achieved.

This study specifically targeted hard-to-reach truckers and, while it resulted in an increased testing rate, the vast majority of truckers in the sample, even in the intervention group, did not test. Additional research is needed to explore the reasons why these hard-to-reach truckers do not test and what additional types of programs might encourage more to test. It could be that in order to reach the hardest-to-reach, a number of different new program options will be needed, with clinic-distribution of self-test kits being only one such program. It is possible that we would have had a greater increase in HIV testing if we had distributed the HIV self-test kits directly to the truckers. Clinic-based distribution may not reach those who are unable to access or do not feel comfortable going to clinics. Other studies that used direct or secondary distribution mechanisms to bring HIV self-test kits directly to potential users found increased testing rates with risk ratios from 1.1 to 2.1 [10–13], which are actually somewhat lower than what we found. However, given the difficulty in reaching some truckers, such as those in this study, direct distribution of self-test kits, coupled with clinic pick-up as an option, might be worth exploring. The North Star Alliance already conducts outreach at truck stops and direct distribution of self-test kits might be added to that program to try to further improve testing rates among the hard-to-reach.

The majority of the study participants in the intervention group who tested chose to self-test (64.5%), but the SOC test was chosen by over a third of participants. Among those who chose to self-test, about two-thirds opted to do so in the clinic with supervision and one-third took a test kit for home use. Thus it seems that people vary in their preferences around HIV testing, which suggests that offering choices may increase the chance that a client can find an acceptable HIV testing option. Similar diversity in preferences was found in our previous study [3, 9], and in a Zambian study [11]. The popularity of self-testing with supervision evident in all these studies warrants further exploration. It could be that some people feel the need for guidance their first time self-testing, and going forward they would instead self-test unsupervised at home, or it could also be that some people’s ideal HIV test would be an oral provider-administered test, and supervised oral self-testing was the closest they could get to that in these studies. A discrete choice experiment we conducted in our previous study among truckers suggested that people have strong preferences regarding type of test (blood versus oral) and form of counseling (in-person versus over the phone) and these preferences differed by HIV testing history; however, preferences regarding who administers the HIV test as well as the testing location were not strong [22]. Thus, future studies might explore different combinations of test choices, such as provider-administered oral tests and self-administered blood tests, to try to determine which tests are the most popular and what the array of testing choices should be in order to maximize testing uptake.

This study had some limitations. Our text message was short and some may not have understood it or were unclear about what a self-test was. This may have weakened the potential impact of the intervention. In addition, the EHRS data may have included errors and misclassifications, causing us to include some who were ineligible, exclude some who were eligible, and misclassify the outcome for some participants. The large number of duplicate phone numbers in the system as well as the missing HIV-testing data on five participants who self-tested suggests a fair amount of error in the system. It is also possible that some may have tested during follow-up at clinics not part of this system and those would be misclassified in our data. However, the proportion choosing the standard HIV test in the intervention group (11/750) is similar to the proportion in the two SOC groups (10/748 and 10/762), suggesting that the standard test may appeal to a consistent proportion of truckers in all groups and therefore it seems likely that the portion testing in other clinics would also be similar due to randomization. Therefore these errors should be non-differential and bias our results towards the null. Finally, our results cannot be generalized to all truckers as we selected a sample meeting specific criteria, including a failure to test for HIV regularly, and from a clinic-based health record system.

## Conclusions

Announcing the availability of HIV self-testing via text message significantly increased the HIV testing rate among a sample of hard-to-reach truckers, but many still did not access testing. Additional research is needed to identify the combination of HIV testing programs that will further increase testing rates in this group.

## Abbreviations

EHRS: Electronic health record system
FIC: Fogarty International Center
NCI: National Cancer Institute
NHBL: National Heart, Lung, and Blood Institute
NIA: National Institute on Aging
NIAD: National Institute of Allergy and Infectious Diseases
NICHD: Eunice Kennedy Shriver National Institute of Child Health and Human Development
NIDA: National Institute on Drug Abuse
NIH: National Institutes of Health
NIMH: National Institute of Mental Health
OAR: Office of AIDS Research
OR: Odds ratio
RR: Risk ratio
SOC: Standard of care
